# Municipal Meat-inspection Legislation1Read at the Thirty-sixth Annual Meeting of the American Veterinary Medical Association, Atlantic City, September, 1901.

**Published:** 1902-01

**Authors:** G. R. White

**Affiliations:** Nashville, Tenn.


### MUNICIPAL MEAT-INSPECTION LEGISLATION, WITH SPECIAL REFERENCE TO THE LAW AND THE RULES AND REGULATIONS OF THE BOARD OF HEALTH OF THE CITY OF NASHVILLE.[1]

By G. R. White, D.V.S.,
NASHVILLE, TENN.

The hopeless task has been assigned me of showing within the limits of this short paper the merits of a most perfect city ordinance as well as the contents of what we consider a complete set of rules and regulations governing the slaughter of animals and the sale of fresh meat in the city of Nashville, together with a few facts relative to municipal meat-inspection legislation.

Many of the members present on this occasion will readily recall the wretched and inefficient system of meat-inspection which confronted them when this Association honored our graceful Southern city by meeting within its confines in the year 1897. Perhaps many of you who are acquainted with the then shameful situation were surprised upon receiving the official programme of this meeting to know that reformation had taken place, and that we were able to come before this meeting with a paper of such a character.

Meat-inspection has been advocated and practised among the Jews and Arabs from time immemorial, and has been one of the principal topics for study and reflection before this Society at each annual meeting during the past decade. It has also been discussed by the different medical organizations throughout the country as well as by boards of health and live-stock sanitary boards until its importance, in fact, its absolute necessity, ceases to be a doubt with the thinking meat-consuming populace of every city.

Since its importance is no longer questioned, then why may we not be permitted to discuss the fundamental principle of a meat-inspection system, viz., legislation? Without proper legislation our efforts are in vain, and nothing can be done toward condemning that meat which is diseased or otherwise unwholesome, and to punish the unscrupulous butcher and meat vendor who are responsible for such flesh being offered for sale as human food. There is no article of food which deserves more attention at the hands of the consumer than this one demands; however, it is surprising how little some people regard and how meagrely the lawmakers

[1] Read at the Thirty-sixth Annual Meeting of the American Veterinary Medical Association, Atlantic City, September, 1901.

of most American cities grasp this important question. Since being associated with the Board of Health of the City of Nashville for the past three years as its meat- and live-stock inspector the writer has obtained actual facts which make him deeply sensible of its importance.

A bit of history of the passage of our meat-inspection law will not be amiss at this immediate juncture.

Since it is the function of the daily press of a city to instruct the people in all branches of science and all forms of knowledge, as well as to gather and condense news for them, thereby advancing their political and social interests, and realizing that by means of the press the public would learn to have more respect for the interested veterinarian and to more fully appreciate the work which the veterinary profession as a whole are constantly struggling so earnestly to accomplish, it behooved us as health guardians interested in matters pertaining to meat-inspection to first secure the indorsement and support of the various papers before inaugurating a movement toward establishing a more protective meat-inspection system. After we had the assurance that both of our influential daily papers favored a more perfect and protective meat-inspection law, and had promised to render us all possible assistance and to do or say nothing detrimental to our efforts in that direction, the bill was drafted without further ceremony, and submitted to the health board for their consideration. The Board of Health at that time consisted of the Mayor, the Chairman of the Board of Public Works and Affairs, and City Health Officer. After mature consideration these gentlemen readily consented to unanimously recommend the bill to the City Council for passage. The Council was "thunderstruck; in fact, almost stampeded, when this bill was introduced by one of its youngest yet most intelligent and progressive members. The bill passed first reading, and was referred to the Sanitary Committee for consideration and recommendation. I presume most of you are slightly acquainted with the average sanitary committee of a municipal council, but for the benefit of those not yet so fortunate as to have received an introduction to this august body of sanitarians I will say that they have about as much idea about sanitation and sanitary problems as a hog has about Sunday. I assure you that the Sanitary Committee of the Nashville Council is no exception to the rule; however, I will say in justice to two of the members that they were above the average and supported our measure from the beginning. Even with their support and influence we had a long and hard fight to secure the indorsement of the other three members. In spite of their opposition they finally consented to yield if the Board of Health would make one or two concessions; this the Board did, and the bill went before the Council at their next regular meeting with the unanimous indorsement of the Sanitary Committee and the Board of Health. After a stormy session of debate and much wire-pulling on the part of some butchers and opposing members, the bill passed the Council with only one dissenting vote. The Mayor approved the same, and it became a law.

About this time the charter of our city was changed by the State Legislature, and one among the many changes was that the Board of Health should consist of three reputable practising physicians appointed by the Mayor instead of the political Health Board which had previously existed.

Three physicians were appointed, and it was then their duty to adopt rules and regulations and to enforce the meat-inspection ordinance in the face of a storm of opposition on the part of butchers and live-stock dealers as well as producers. The following is the law which we succeeded in having passed, and I have yet to see a meat-inspection ordinance on the statute books of any American city which is its equal or superior.

### City Ordinance Regulating the Slaughter of Animals and Sale of Fresh Meat.

*Be it Enacted by the Mayor and City Council of Nashville:*

Section 1. That all ordinances and regulations heretofore passed and now in force for the inspection of meat be and the same are hereby repealed, and the following are adopted in lieu thereof:

Sec. 2. That the City Board of Health is hereby invested with the same inquisitorial power and authority to inspect in any place, public or private, any cattle, sheep, hogs, lambs, or veal calves for sale for slaughtering purposes, or that have been slaughtered, for one mile beyond the corporate limits as they possess and can exercise within the city.

Sec. 3. That from and after the passage of this act it shall be unlawful for any person, firm, or corporation to slaughter, sell, or offer to sell the meat of any animal not considered "game," intended for human food, within the city of Nashville, unless the same has been inspected and approved by the officers appointed and empowered by the City Board of Health, or unless such meat bears the mark, stamp, or tag indicative of inspection by the Bureau of Animal Industry, United States Department of Agriculture: *Provided*, however, that this section shall not be construed to prohibit producers residing beyond the limits herein provided from killing animals and selling the same within the city; but no such animals or parts of animals shall be sold until the same shall have been inspected by the inspector in such places as may be designated by the City Board of Health.

SEC. 4. That such meat when so inspected and approved shall be marked, stamped, or tagged by inspectors of said City Board of Health, and the same shall not be allowed upon the stalls of any market, whether public or private, unless so stamped, marked, or tagged, after having been inspected and approved by inspectors of the Board of Health. All tags shall be kept for thirty (30) days from date of issuance, which tags shall bear date of inspection and be at any time subject to examination by the meat and live-stock inspector or by any police officer of the city of Nashville; and it shall be unlawful for any meat dealer to remove the official tag, mark, or stamp until after the meat is sold to the consumer, and no tag, mark, or stamp shall again be used.

SEC. 5. That any person, firm, or corporation who shall counterfeit or knowingly alter, deface, or destroy any of the tags, marks, or stamps of the City Board of Health, shall, upon conviction, be fined not less than ten ($10) dollars and not more than twenty-five ($25) dollars, and, in default of payment, by imprisonment for not less than ten (10) days and not more than thirty (30) days for each offence.

SEC. 6. That the Nashville Abattoir and yards in connection therewith, or such other places as may from time to time be fixed by the City Board of Health, are hereby designated as places at which animals and meats to be used for human food within the city of Nashville and within its police jurisdiction are to undergo an ante-mortem and a post-mortem inspection, and there be tagged, marked, or stamped by the meat and live-stock inspector, except such meat as is provided for in the latter clause of Section 3.

SEC. 7. That this ordinance shall not in any manner apply to the sale of salt, pickled, smoked, or canned meats of any kind.

SEC. 8. That not more than the following prices may be charged and collected by the person, firm, or corporation who now are or who may hereafter be operating the Nashville Abattoir, or such other places as may be fixed by the Board of Health, for slaughtering animals intended for human food within the city of Nashville, to wit: cattle, fifty (50) cents; veal calves, twenty-five (25) cents; hogs, sheep, and goats, fifteen (15) cents; for cold storage: cattle, fifty (50) cents; veal calves, hogs, sheep, and goats, fifteen (15) cents.

SEC. 9. That all animals intended for human food in the city of Nashville and within its police jurisdiction shall undergo an ante-mortem examination before being allowed to pass to the slaughter-room, and a post-mortem examination on the floor of the slaughter-room. All animals, carcasses, or parts of carcasses found on inspection to be diseased or unwholesome from any cause shall be condemned and marked in such a manner as the inspector deems best to indicate its condemnation. All condemned animals, carcasses, or parts of carcasses shall be disposed of otherwise than for food in such manner as the inspector directs.

SEC. 10. That to carry out the provisions of this ordinance the City Board of Health are hereby authorized to appoint a meat and live-stock inspector, at a salary not to exceed one hundred ($100) dollars per month, who shall be subject to removal for cause. The inspector shall give bond in the sum of two thousand ($2000) dollars for the faithful performance of his duties, and make a monthly report of all his official acts in the inspection of meats and live-stock to the City Board of Health, and a monthly report to the Board of Public Works of all professional services rendered the sick and disabled stock of the various departments.

SEC. 11. That the meat and live-stock inspector shall be a qualified veterinarian, a graduate of an organized and recognized veterinary college, and of good standing in his profession.

SEC. 12. That the meat and live-stock inspector is hereby clothed with police powers, and shall wear a badge while performing his official duties.

SEC. 13. That the meat and live-stock inspector shall hold an ante-mortem and a post-mortem examination of all animals intended for human food within the city of Nashville or within its police jurisdiction, and shall tag, mark, or stamp such animals as in his judgment are suitable for human consumption. He shall visit from time to time the various meat markets of the city and examine all meat therein, to see that same is properly tagged, marked, or stamped. He shall render all required professional services to the sick and disabled live-stock of the various departments. He shall investigate all contagious or infectious diseases which are reported to the City Board of Health, as is provided for in an act of the City Council, entitled "An Ordinance to prevent the spread of communicable diseases among domestic animals in the city of Nashville."

SEC. 14. That the City Board of Health shall have the power and authority to adopt such rules and regulations as may from time to time become necessary to carry out the provisions of this ordinance.

SEC. 15. That the person, firm, or corporation violating any of the provisions of this ordinance, or any of the rules and regulations adopted by the City Board of Health, as is provided in Section 14 hereof, shall upon conviction be fined not less than ten ($10) dollars nor more than twenty-five ($25) dollars for each offence. Each animal, carcass, or part of carcass slaughtered or sold in violation of this ordinance shall be considered a separate offence.

SEC. 16. That this ordinance shall take effect from and after its passage, the welfare of the city requiring it.

Passed July 27, 1899.

Approved July 31, 1899.

R. H. DUDLEY, Mayor.

Attest : J. P. Byrne, Recorder.

In compliance with the above ordinance the following rules and regulations were adopted by the Board of Health :

### RULES AND REGULATIONS FOR THE INSPECTION OF LIVE-STOCK AND FRESH MEAT.

By virtue of the authority conferred upon the Board of Health of the city of Nashville, under the provisions of an ordinance passed July 27, 1899, and approved July 31, 1899, entitled "An Ordinance regulating the slaughter of animals and the sale of fresh meat," the following rules and regulations are hereby prescribed :

1. Slaughter houses, except those specified in the above-mentioned ordinance, which are designated as places for ante-mortem and post-mortem inspection, shall be constructed according to the plans and specifications of the Board of Health. And it is further ordered that all animals slaughtered and all meats tagged and stamped under the supervision of the inspector of the United States Department of Agriculture, at any packing house, will meet the requirements of these rules.

2. All livers, lungs (lights), spleens (melts), and tongues of all animals slaughtered shall be hung on racks provided for that purpose, immediately after the slaughtering and removal from the carcasses of the animals, and shall there remain until the inspector of the Board of Health shall have examined and inspected the same, and shall not be removed therefrom except by permission of said inspector ; and all such organs shall be marked by the butchers on placing them on the rack in such manner that the said organs can be easily identified with the carcasses from which they have been removed.

3. At least one inch of the diaphragm or skirt of all carcasses of slaughtered animals shall be left on the animal slaughtered until the meat-inspector shall have examined, inspected, and passed the same, and all the parietal pleura, or the lining of the chest cavity, and the parietal peritoneum or casing of the abdominal cavity, ordinarily removed in the process commonly known as "stripping," shall be allowed to remain on the carcass and shall not be removed therefrom by the processs known as "stripping" until after examination by the inspector.

4. No person shall urinate, defecate, or commit any nuisance whatsoever in the building or buildings, or on the premises adjacent thereto, except in places properly provided for that purpose.

5. All slaughtering and dressing of animals shall be completed, and all offal, refuse, horns, etc., shall be removed daily by 5.30 P. M., and the tubs, buckets, or other receptacles in which they are deposited shall be cleansed and disinfected from time to time, as the meat and live-stock inspector may direct, and the floors, walls, etc., of the slaughtering rooms shall be flushed, washed, and thoroughly cleansed each day.

6. The hours for inspection at all establishments designated as places for inspection shall commence at seven (7) A. M. and continue until five-thirty (5.30) o'clock P. M.

7. In the event of rejection or condemnation of any animal by the inspector, permission is hereby granted the owner of such animal so rejected or condemned to slaughter another one, in order to replace same, even after the hour above mentioned; provided the inspector has due notification by the owner of his intention to slaughter another animal to replace the rejected or condemned one, and it shall be the duty of the inspector to remain until said animal is dressed and inspect same, and, if approved, said carcass shall be marked, stamped, or tagged.

8. The owner of a condemned animal or carcass shall have the right to view same before it is disposed of, provided he does so within twenty-four (24) hours after it is condemned.

9. All carcasses or parts of carcasses consigned to the rendering tank shall be "tanked" in the presence of the official inspector of the Board of Health.

10. All meat markets or shops, and all refrigerators or ice-boxes or other places where fresh meat is stored or handled, and all wagons used in the transportation of fresh meat, shall be kept in such sanitary condition as the Board of Health or the meat and live-stock inspector may approve.

11. No carcass or part of carcass shall be allowed to hang on the outside of markets, shops, or stores exposed in such a manner as to become contaminated by dust or dirt.

12. All carcasses or parts of carcasses sold, handled, or delivered from wagons or other vehicles shall be kept covered in such manner as to prevent contamination by dust or dirt.

13. All carcasses or parts of carcasses offered for inspection by the producer shall be hung on hooks provided for this purpose in the north end of the market house, and there remain until inspected and approved by the meat and live-stock inspector.

14. Each carcass or part of carcass offered for inspection by the producer shall be accompanied by the lungs (lights), liver, heart, kidneys, spleen (melt), and tongue.

15. The lungs (lights), liver, heart, and kidneys of all animals, except cattle, shall be attached to the carcass in such manner as to identify the animal to which they belong.

16. The lungs (lights), liver, heart, and kidneys of cattle shall accompany the carcass and be certified by the owner or person in charge of same as being those removed from the carcass or part of carcass then undergoing inspection.

17. All carcasses or parts of carcasses offered for inspection by the producer shall be inspected under the same conditions and limitations as are provided in Rule 3.

18. The hours for the inspection of producers' meat shall begin at 6 A. M. and 4 P. M. and continue until such inspection is completed.

19. In special cases where, in the opinion of the meat and live-stock inspector, the circumstances or necessities of individual cases warrant the same, that officer may grant special permits to dispense with the observance of any of the foregoing provisions.

20. All tags, seals, stamps, and other devices used in marking, stamping, or tagging animals and dressed carcasses shall be in possession of the meat and live-stock inspector, and used by him or under his supervision or direction. The inspector in charge of each place of inspection is instructed to use such safeguards as in his judgment are necessary to account for every tag and seal issued to them, and to have the work of affixing so carefully supervised that nothing but inspected meat will bear the official mark, stamp, or tag of the Board of Health of the city of Nashville.

21. The inspector, in determining what constitutes a diseased animal or meat unwholesome and unfit for consumption, shall be guided by the specifications contained in the regulations of the United States Department of Agriculture.

22. A copy of these rules and regulations shall be printed and kept wherever fresh meat is inspected, stored, or offered for sale in the city of Nashville or within its police jurisdiction.

NOTE. The above rules were adopted by the City Board of Health, in regular meeting, on February 25, 1901.

The aforementioned law, in conjunction with the above rules and regulations, gives the Board of Health absolute control of the meat-supply of our city.

We are now confronted with the yet unanswered problem why all municipalities are not able to establish and maintain a protective system of inspection of their meat-supply. This I hope Dr. C. A. Cary, who has just preceded me with his most excellent paper, has answered to your entire satisfaction. In my opinion, "politics" is the greatest obstacle to be overcome. It is to be presumed that many of the members present on this occasion have had more or less experience before State Legislatures and City Councils in their endeavor to secure legislation for the protection of the unsuspecting public. Those of you who have had such experience appreciate what "high life before a legislative body" means, and how difficult it is to secure their co-operation and support in the adoption of any meritorious health measure. Certain scientific facts seemingly inherent to the average "law-maker" are well known and their importance duly appreciated by the educated and conservative members of the veterinary profession, and especially by the oldest and most experienced members of the Association. Many of you no doubt have long since concluded that if any human being or set of human beings are capable of doing the impossible it is a "ward politician" when he becomes a member of a "law-making body" of a municipality.

Apparently the veterinary profession of America during its years of existence has had but little if any perceptible influence in formulating the fundamental laws governing the inspection and sale of meat and milk in the majority of American cities. This is a remarkable phenonnon in our professional history—remarkable because no intelligent or scientifically educated gentleman in possession of the facts disputes the ability and integrity of the great majority of the members of the veterinary profession; and it is still further remarkable when we consider the great number of our profession, laboring incessantly for the enactment of laws for the protection of public health, who do not seek, nor do they desire, political office or distinction. In my opinion, the explanation of this singular situation is to be found in the fact that the veterinary profession and this Association of veterinarians have in the past failed to concentrate and direct their educative efforts and influence to the right source. I said apparently the veterinary profession of the country had exercised but little influence toward shaping meat and milk-inspection laws in the majority of American cities; I repeat it, emphasizing the word *apparently*, as I am not now dealing with the direct or the indirect influence of individual members of the veterinary profession, but am speaking of the profession as a collective body in having legislation enacted. The thirty-eight years of life of this organization, and its ever-recurring failure to induce our National Congress, State Legislatures, County Courts, and Municipal Councils to pass its proposed measures, demonstrates the necessity for different methods of procedure from any which have been advocated in the past if this Association expects to have its proposed measures enacted into laws. A striking example of our recommendations being ignored, and collapse of our hopes, will be found in the fate of the recently proposed army veterinary legislation, and so it is with many other propositions from the veterinary profession.

As a general proposition, if the lessons of the past prefigure a truth of the future it will be a useless waste of time, as well as an invitation to the worry and vexation of disappointment, to go before the "law-makers" with our measures unless we first get the indorsement and co-operation of influential citizens, their masters—people upon whose votes and influence they depend for indorsement of their official acts and re-election.

There is an inseparable connection between veterinarians and veterinary sanitary laws, and it is the duty of our profession to stamp its impress upon legislation of the nation, State, county, and municipality, to the end that the veterinary profession may not suffer in the estimation of the public by the passage of bad or inferior laws which have in them no real merit or force.

We should take such an interest in the passage of good and wholesome legislation as will inspire the public with confidence in our integrity, ability, and professional interest in matters pertaining to public health. Veterinarians are in a large measure held responsible, and sometimes unjustly censured, for all municipal meat-inspection legislation, whether it be good, bad, or indifferent. Since this is true, then let us, as a glorious and time-honored profession which many noble and scholarly men and women respect and admire, set our heel firmly upon the neck of the political shyster as a law-maker and the "butcher" as a competent meat-inspector, thereby to a certain extent preventing them from bringing reproach upon a profession held most dear to all present on this occasion as well as every veterinarian who appreciates his profession.

I will close with the admonition that when you have succeeded in gaining the confidence of the people, and have made your impress upon them, then, and only then, will there be developed the proper relationship between our profession and the municipal law-maker; and you will at that time be able to accomplish something toward veterinary sanitary legislation that will be a protection to the public as well as of lasting and permanent benefit to the veterinary profession.

